# Maritime Freight Carbon Emission in the U.S. using AIS data from 2018 to 2022

**DOI:** 10.1038/s41597-024-03391-0

**Published:** 2024-05-25

**Authors:** Cheng Cheng, Zengshuang Li, Yuting Yan, Qiang Cui, Yong Zhang, Lei Liu

**Affiliations:** 1 School of Transportation, Southeast University, Nanjing, 211189 China; 2 Key Laboratory of Transport Industry of Comprehensive Transportation Theory (Nanjing Modern Multimodal Transportation Laboratory), Ministry of Transport, Beijing, P. R. China; 3 Yangtze River Delta Carbon Neutrality Strategy Development Institute, Southeast University, Nanjing, 211189 China; 4 School of Economics and Management, Southeast University, Nanjing, 211189 China

**Keywords:** Environmental impact, Environmental impact, Marine biology

## Abstract

Global maritime emissions, a 3% contributor to greenhouse gases, anticipate a surge of 90–130% by 2050. Regulatory challenges persist due to international governance gaps. Legislative strides, including the EU Emission Trading System, highlight global efforts. In the U.S., despite legislative commitment, consensus hurdles impede cross-regional carbon management. Prevailing top-down emissions estimation methods warrant scrutiny. This paper unveils U.S. maritime emissions intricacies, focusing on carbon accounting, transfer, and compensation for cargo and tanker vessels. Leveraging AIS data (2018–2022), an activity-based/bottom-up approach navigates emissions calculations, aiming to reshape understanding and foster strategic reductions. The study bridges gaps in U.S. maritime emission research, promising insights into transfer and compensation dynamics. By concentrating on high-impact vessel types, it contributes to emissions mitigation strategies, steering towards a sustainable U.S. maritime future.

## Background & Summary

The maritime sector has emerged as the swiftest-growing contributor to greenhouse gas (GHG) emissions globally, accounting for approximately 3% of the total and experiencing a 20% increase over the last decade^1^. With the escalating demand for maritime transport, emissions are anticipated to surge by 90–130% by 2050 compared to 2008^2^, contradicting the temperature control objectives of the Paris Agreement and necessitating immediate emission reductions in maritime transportation. However, the international nature of maritime transport keeps it beyond the scope of the Paris Agreement, lacking governmental control and forming a regulatory gray area for carbon emission reduction.

In January 2023, the EU’s legislative bodies reached an agreement to integrate shipping into its Emission Trading System (EU ETS). Pending final adoption by the EU, ships with a gross tonnage (GT) above 5000 engaged in the commercial transport of cargo or passengers within the EU will be obligated to obtain and surrender emission allowances for their CO_2_ emissions starting from 2024^3^.

The United States, endowed with a well-established maritime transportation system, actively participates in carbon reduction initiatives within the maritime transport sector. Proposed legislations, such as the “U.S. Proposes Legislation to Tax Marine Carbon Fuels and Port Emissions,” exemplify these efforts. Nevertheless, achieving consensus on crucial aspects of cross-regional carbon management or taxation in maritime transport proves to be a challenge. Disputes persist over responsibilities for greenhouse gas emissions and compensation mechanisms for affected parties^4^.

The assessment of maritime carbon emissions is of paramount importance in the realm of global environmental stewardship. Esteemed organizations such as the International Maritime Organization (IMO), European Environment Agency (EEA), and U.S. Environmental Protection Agency (EPA) have delineated two fundamental methodologies for estimating shipping air pollutants: the fuel-based approach, relying on direct observations, and the activity-based approach, integrating statistical analyses of activity data with country-specific emission factors^5,6^. The former represents a top-down methodology, calculating emissions without spatial specifics^7–9^, while the latter, a bottom-up strategy, utilizes diverse and comprehensive data sources, especially ship trajectory data such as AIS (Automatic Identification System) information^7,10^.

Traditionally, the top-down approach has been widely employed in emission inventories^11,12^, providing a broad overview of emission trends at the macro level. However, it disregards specific differences involving ship type, route, and trajectory features, leading to accuracy issues. Moreover, this approach fails to provide a detailed inventory of emissions, hindering accurate assessments of emissions and transfers from specific ships or individual voyages. Consequently, the top-down approach is limited in accuracy, timeliness, and effectiveness, potentially failing to meet the increasing demand for environmental regulation and regional emission reduction.

In contrast, the activity-based/bottom-up approach is more feasible, albeit challenging in data acquisition and necessitating data preprocessing. Through the collection and analysis of ship-specific activity data, this approach can more accurately reflect the carbon emissions and transfer of each vessel. Recently, with the increasing availability of detailed ship trajectory data, the bottom-up approach has gained prominence, becoming the primary method for assessing ship exhaust emissions. A pivotal technique within this approach is the STEAM (Ship Traffic Emissions Assessment Model) method^13^, extensively applied in various regions, including the Ports of Los Angeles and Long Beach^14^, Shenzhen port^15,16^, and the Yangtze River^17^. This method not only calculates emissions at regional levels but also contributes significantly to understanding the spatially distributed carbon footprint across various scales, from ports to global analyses^18–20^. The comparison between the top-down and bottom-up methods is presented in Table 1.

**Table 1:** The comparison between the top-down method and bottom-up method.

Aspect	Top-Down Method	Bottom-Up Method
Definition	Estimates emissions based on aggregated data and models	Calculates emissions based on detailed operational data
Data Requirement	Requires global data on vessel activity and fuel usage	Requires vessel-specific operational data
Accuracy	Provides rough estimates, prone to inaccuracies	Offers higher accuracy by accounting for specific vessel activities
Complexity	Less complex, relies on statistical models	More complex, involving detailed data collection and analysis
Granularity	Offers high-level insights into overall emissions	Provides detailed insights into emissions per vessel
Applicability	Suitable for large-scale analysis and policy planning	Suitable for regional analysis and policy planning, individual vessel emissions and performance
Cost	Generally lower cost due to reliance on aggregated data	Can be costlier due to the need for detailed data collection and analysis
Uncertainty	Prone to uncertainties due to reliance on aggregated data	Can reduce uncertainties by directly measuring emissions from vessels
Compliance Verification	Less effective for verifying compliance with regulations	Effective for verifying compliance and identifying areas for improvement
Regulatory Alignment	May lack alignment with specific regulatory requirements	Can align closely with regulatory reporting requirements

Despite advancements in maritime emission assessment methods, research on carbon emissions transfer and compensation within the maritime transport sector remains relatively underexplored, especially in the United States. Existing studies have predominantly focused on provincial-level carbon emission transfers^21–25^ and spatio-temporal evolution of carbon transfer^26,27^ in China with limited exploration of carbon transfer dynamics in the U.S. maritime industry. Moreover, existing research on carbon compensation primarily addresses overall compensation between regions^28–30^, often overlooking the specific intricacies of the maritime industry.

This paper aims to bridge these gaps by delving into carbon transfer and compensation within the maritime shipping industry in the U.S. Additionally, we will focus on cargo and tanker vessels since cargo carriers and tankers contributed more than 80% of the total maritime carbon emissions^20^. Utilizing AIS data spanning from 2018 to 2022, this study endeavours to rejuvenate and expand the realms of carbon emission transfer and compensation research. Through this exploration, the paper intends to offer valuable insights, significantly contributing to the understanding of maritime emissions and providing practical recommendations for promoting emission reduction strategies within the U.S.‘s maritime sector.

**Fig. 1:**
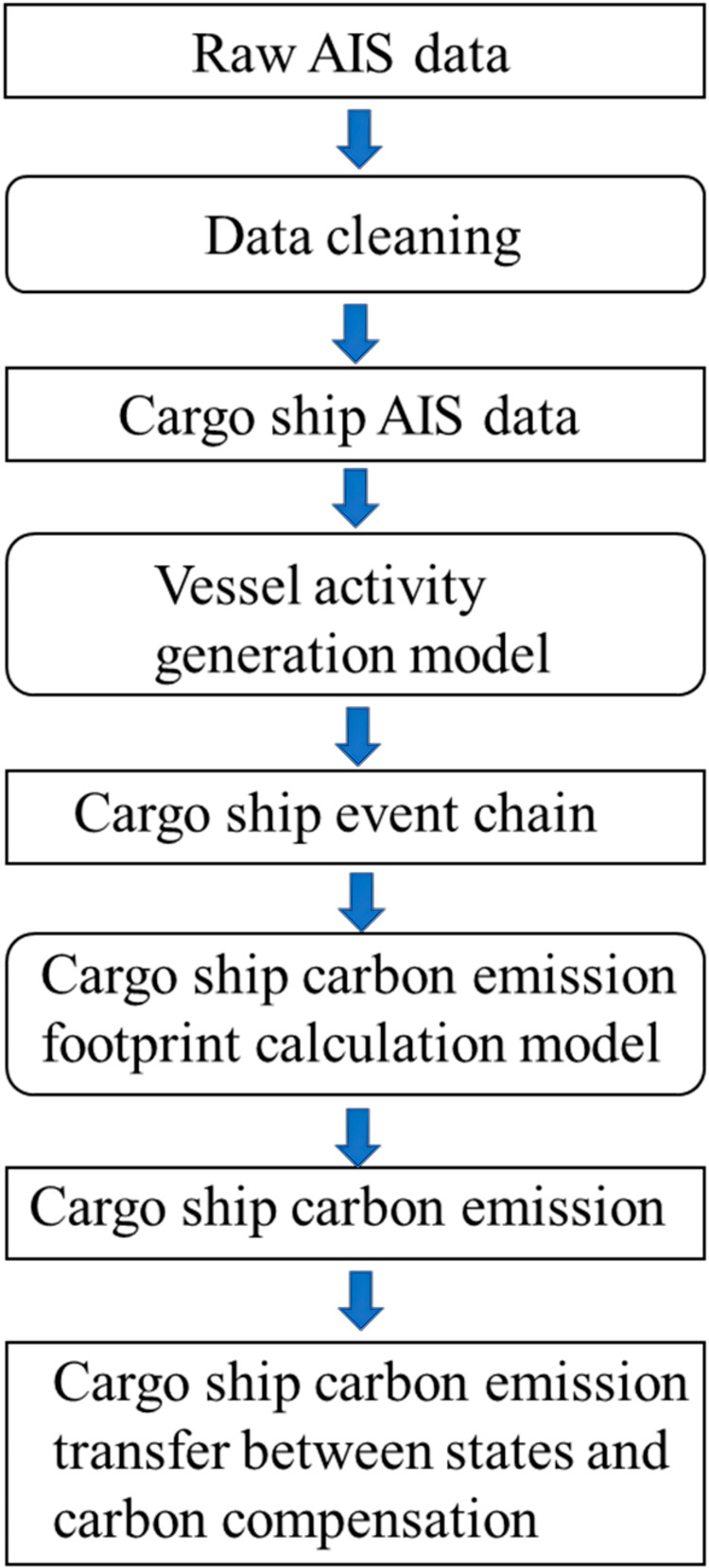
Workflow of this study.

This paper will apply an activity-based/bottom-up method using AIS data to calculate the carbon footprint of cargo ships visited U.S. coastal waters. AIS data offers unparalleled granularity, providing high-frequency vessel movement data, allowing for a detailed understanding of the temporal and spatial dynamics of maritime activities. Importantly, AIS data has been available over an extensive period, offering a longitudinal perspective that is invaluable for tracking trends and changes in maritime emission. Unlike traditional top-down approaches, AIS facilitates a bottom-up methodology, enabling precise carbon emission calculations for individual vessels. This data-driven approach not only enhances the accuracy of emission assessments but also allows for a comprehensive analysis of carbon transfer and compensation dynamics. The flow chart of the current paper is presented in Fig. 1. The methodology involves data collection, cleaning, and the development of a vessel activity model to understand event chains. Precise carbon emission calculations are conducted for individual vessels, forming the basis for in-depth analysis of carbon transfer and compensation dynamics within the maritime industry. This research aims to provide comprehensive insights into maritime carbon emissions, shedding light on transfer patterns and compensation mechanisms in the U.S. context.

## Methods

### Collect AIS data

**Fig. 2:**
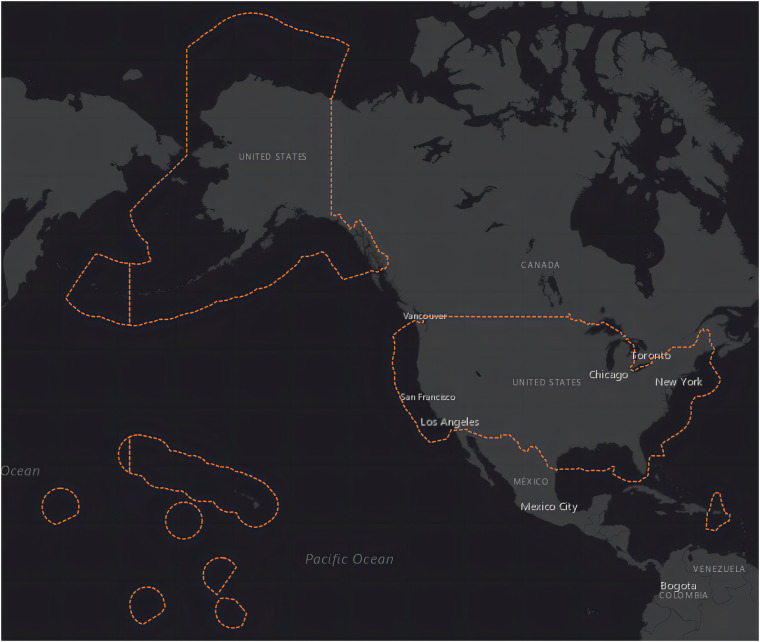
Data collection area (derived from Vessel Traffic Data website^31^).

The AIS data utilized in this study were sourced from the Vessel Traffic Data website^31^ (MarineCadastre.gov | Vessel Traffic Data), made available by the U.S. Coast Guard. This data is derived from onboard navigation safety devices, providing real-time monitoring of vessel locations and characteristics within U.S. and international waters. The data collection area is visually depicted in Fig. 2, which covers important navigation routes in the conterminous U.S., Alaska, Hawaii, Guam, and parts of the Caribbean. To obtain the AIS data, users are directed to the “AIS Broadcast Point button,” where clicking it leads to the AIS data file. Each year’s data is consolidated into a single file, with individual days’ data accessible upon selecting the respective year. Downloading each day’s data individually was a time-intensive process, spanning from 2018 to 2022, constituting a crucial aspect of this research effort. The attributes of the AIS data we downloaded are shown in Table 2. The ones used in the current research are MMSI, BaseDateTime, LAT, LON, SOG and Vessel Type.

**Table 2:** The attributes of the data which are included in the downloaded AIS data^31^.

	Name	Description	Example	Units	Resolution	Type	Size
1	MMSI	Maritime Mobile Service Identity value	477220100			Text	9
2	BaseDateTime	Full UTC date and time	2017-02-01T2005:07		YYYY-MM-DD:HH-MM-SS	DateTime	
3	LAT	Latitude	42.35137	decimal degrees	XX.XXXXX	Double	8
4	LON	Longitude	−71.04182	decima degrees	XXX.XXXXX	Double	8
5	SOG	Speed Over Ground	5.9	knots	XXX.X	Float	4
6	COG	Course Over Ground	47.5	degrees	XXX.X	Float	4
7	Heading	True heading angle	45.1	degrees	XXX.X	Float	4
8	Vessel Name	Name as shown on the station radio license	OOCL Malaysia			Text	32
9	IMO	International Maritime Organization Vessel number	IMO9627980			Text	7
10	Callsign	Call sign as assigned by FCC	VRME7			Text	8
11	Vessel Type	Vessel type as defined in NAIS specifications	70			Integer	short
12	Status	Navigation status as defined by the COLREGS	3			Integer	short
13	Length	Length of vessel (see NAIS specifications)	71.0	meters	XXX.X	Float	4
14	Width	Width of vessel (see NAIS specifications)	12.0	meters	XXX.X	Float	4
15	Draft	Draft depth of vessel (see NAIS specifications)	3.5	meters	XXX.X	Float	4
16	Cargo	Cargo type (see NAIS specification and codes)	70			Text	4
17	Transceiver Class	Class of AIS transceiver	A			Text	2

### Data cleaning

**Fig. 3:**
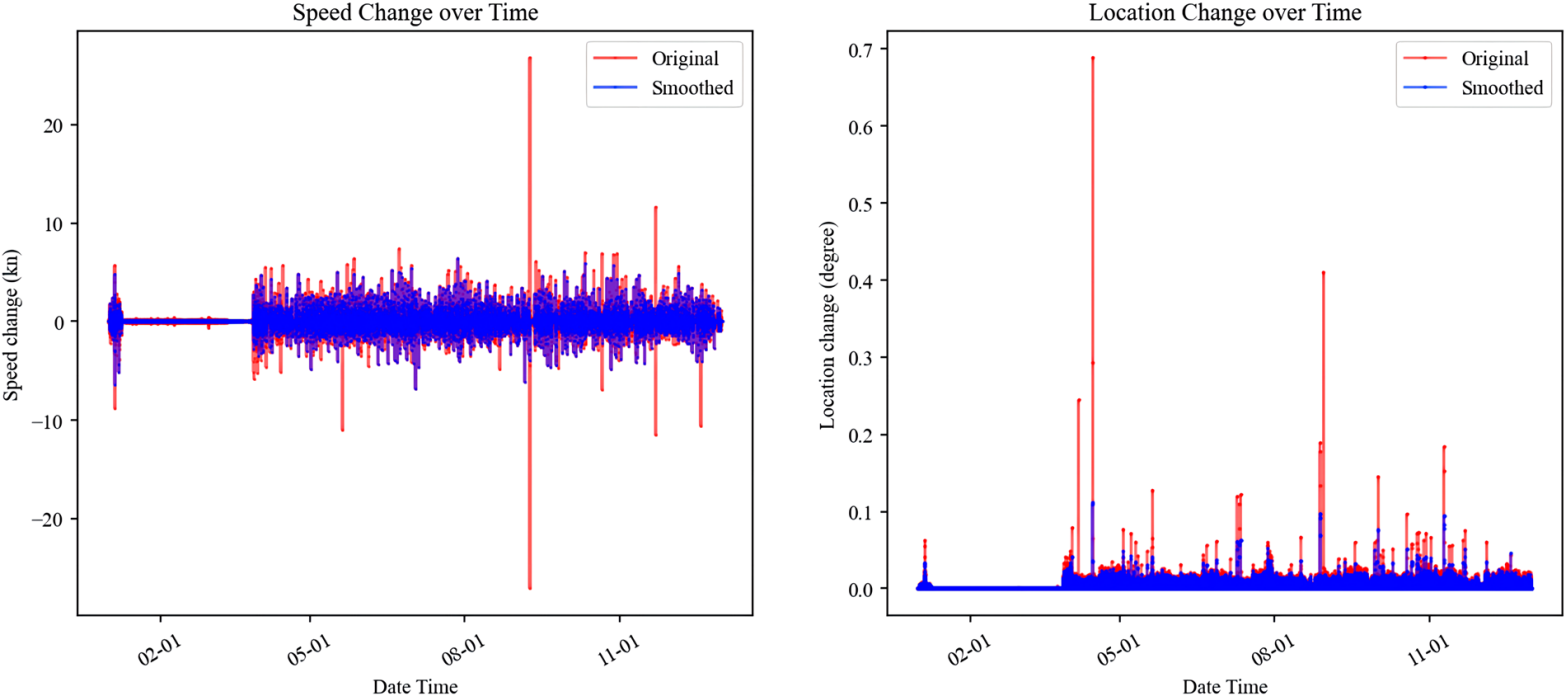
Distribution of speed change and location change from AIS data.

**Fig. 4:**
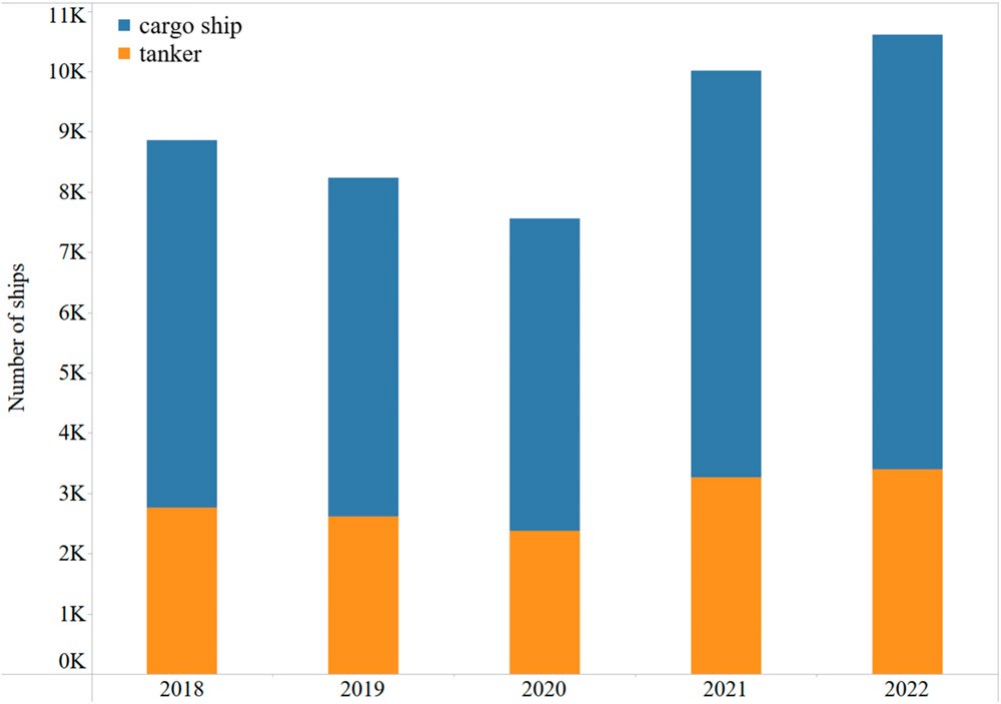
Number of vessels from 2018 to 2022.

The original dataset comprised AIS data from various vessels, with our primary focus directed toward cargo and tanker vessels. Filtering was implemented based on the “VesselType” attribute, wherein each vessel received a numerical code cross-referenced with a Vessel Group table. This table categorized vessels into nine groups, specifically identifying cargo vessels as “VesselTypes” 70 to 79 and tankers as “VesselTypes” 80 to 89. Subsequently, a preliminary data analysis was conducted to identify anomalous records. By grouping the data using “MMSI,” the trajectory of each vessel was established through the ranking of data entries according to “BaseDateTime.” Speed changes and distances travelled between consecutive records, utilizing attributes such as “BaseDateTime,” “LAT,” “LON,” and “SOG,” exposed anomalies as depicted in Fig. 3. This figure presents example plots illustrating the speed change and distance change over time for a specific vessel. The visualizations indicate that aberrant records were effectively mitigated using a median filter, resulting in the refinement of the dataset. Following this, the data is prepared for calculating the carbon emissions of freight shipping. The number of vessels recorded in different years is depicted in Fig. 4, with the valley in 2020 potentially attributed to the outbreak of COVID-19 at that time. The past two years have seen a noteworthy increase, with approximately 10,000 vessels recorded in 2022.

### Calculate the carbon emission

**Fig. 5:**
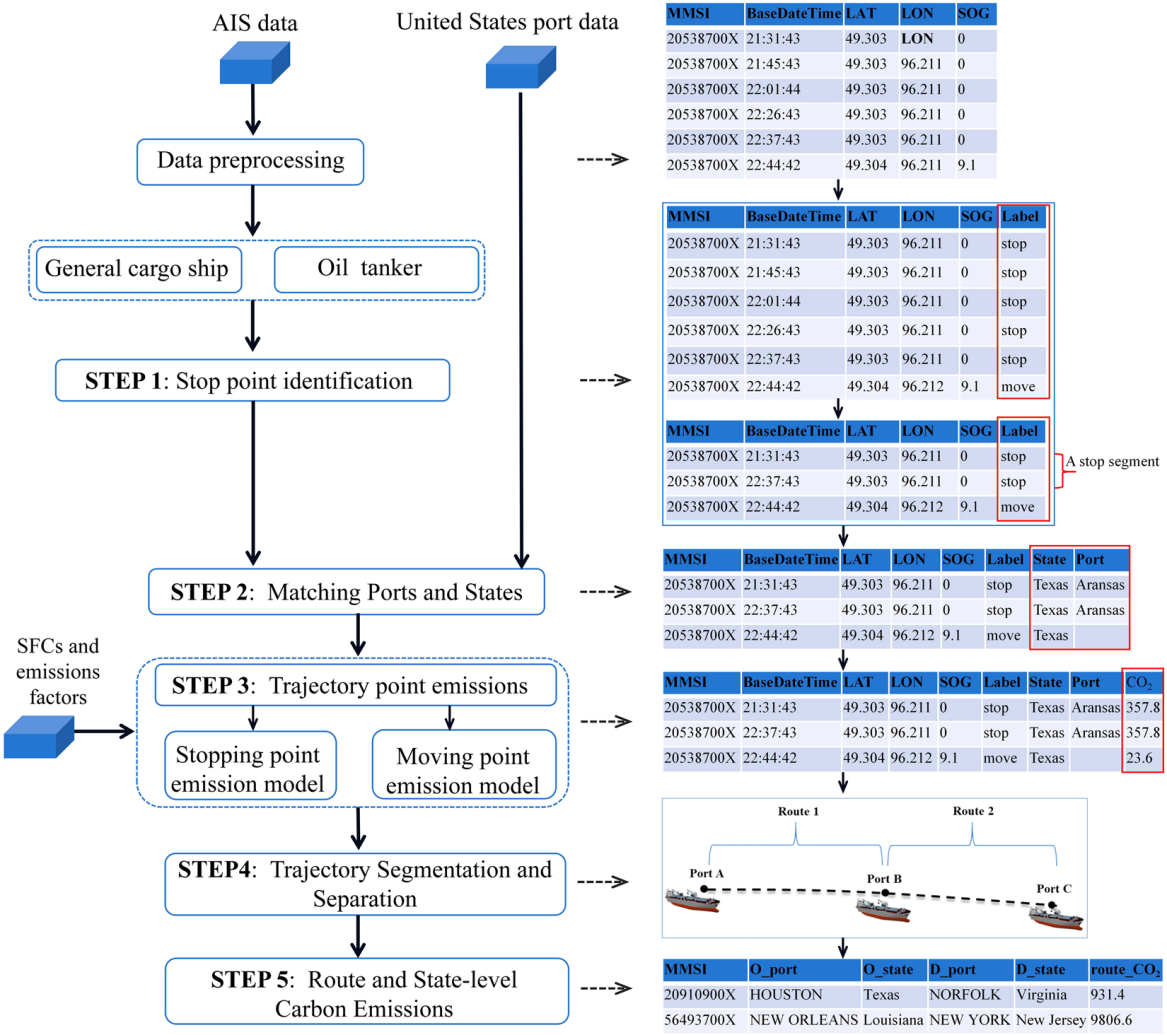
Carbon emission calculation process.

The carbon emissions from cargo and tanker vessels are determined using a bottom-up, activity-based approach, which is divided into five key steps as illustrated in Fig. 5. The fundamental steps of the emission calculation framework are outlined below:

Step 1: Stop Points Identification

In the initial step, we developed a vessel activity generation model to produce event-chains for vessels based on trajectory data processed through data cleaning. The primary objective of this activity generation model is to differentiate between vessels’ moving and stationary activities, a crucial distinction in carbon emission calculation. The algorithm identifies potential stops by evaluating vessel speed, categorizing and marking trajectory points with speeds below 0.3 knots as potential stopping points. The continuous stopping points form potential stay segments. Subsequently, the start and end indices of the stay segment are identified. For each potential stay segment, if the duration exceeds a defined threshold, the algorithm designates the corresponding trajectory segment as an actual stopping segment. If the time difference between consecutive stay segments is below a predetermined threshold, indicating continuous stationary periods, the algorithm merges the two stay segments into a single stopping segment. Next, the algorithm identifies the start and end indices of each stopping segment, and removes the rows between these indices. Other trajectory points are considered as moving points. The output at this step is a labelled dataset, with all trajectory points marked with stop or move labels.

Step 2: Matching Ports and States

This step aims to match each trajectory point with its geographical information. For each stop point, we utilize the pre-obtained USA port database to link it to the nearest port and its corresponding state based on geographical coordinates. Similarly, we identify the state for each movement point, where available within U.S. territorial sea boundaries, in preparation for accounting for carbon transfers. We exclusively account for stops at ports within U.S. territorial boundaries in our carbon emissions calculations. This strategy ensures that our analysis accurately mirrors the environmental impact of shipping activities directly linked to U.S. ports, while omitting transit emissions that do not relate to U.S. port activities. The output at this step is a dataset appended with the port (if any) and state where the trajectory point is located.

Step 3: Trajectory Point Carbon Emissions Calculation

In the third step of our methodology, we employ distinct equations to quantify carbon emissions for vessel movement and stop activities individually. For vessel movement, carbon emissions are computed using Eq. (1), wherein the calculation is grounded in fuel consumption and the emission coefficient. Previous research has underscored that the fuel consumption rate during a ship’s motion is primarily associated with factors such as sailing distance, speed, and engine power^32–34^. Importantly, this rate appears to be nearly independent of displacement, cargo weight, and wind direction^35,36^. Consequently, our calculation exclusively incorporates engine power to determine fuel consumption, given its substantial influence on emissions during vessel movement.

The Emissions from ship movement E_ij_ can be calculated by

1 $$\begin{array}{lll}{E}_{ij} & = & {I}_{k}\times F{C}_{ij}\\  & = & {I}_{k}\times \frac{{S}_{ij}}{\overline{{v}_{ij}}}\times SFC\times {p}_{s}\times 1\times 1{0}^{-6}\,{\rm{tonnes}}\end{array}$$

*I*_*k*_ is the emission coefficient of pollution k of ship residual oil; *FC*_*ij*_ is the fuel consumption of the ship moving from trajectory point *i* to *j*; *S*_*ij*_ is the distance from trajectory point *i* to *j*, measured in nautical miles; $\overline{{v}_{ij}}$ is the average speed from trajectory point *i* to *j*, measured in knots; *p*_*s*_ is the engine power of the ship. According to the classification method of ships in the U.S. AIS data, we take *p*_*s*_=9300 *kW* for general cargo ships and *p*_*s*_=9400kW for oil tankers^37^; SFC is the specific fuel consumption, with SFC=213.1 g/kWh^38^.

For CO_2_, the emission coefficient is fixed, which is ${I}_{C{O}_{2}}=3.114$ tonnes/tonnes^39^.

For vessel stop activities, Eq. (2) is employed to compute carbon emissions. The fuel consumption during the stationary phase is primarily associated with the auxiliary engine power and the duration of the stay. Following the auxiliary engine fuel consumption calculation method proposed by Colling, A.^40^, the emissions from the ship during the stay stage can be determined by:

2 $$\begin{array}{lll}{E}_{stop} & = & {I}_{k}\times F{C}_{stop}\\  & = & {I}_{k}\times {p}_{a}\times {t}_{stop}\times SFC\times 1\times 1{0}^{-6}\,{\rm{tonnes}}\end{array}$$

*FC*_*stop*_ is the fuel consumption generated by the ship while staying in the port; *t*_*stop*_ is the time of port call of the current ship; we take *p*_*a*_=1776 kW for general cargo ships and *p*_*a*_=1985 kW for oil tankers^37^.

The output at this step is a dataset appended with carbon emissions for each trajectory point.

Step 4: Segmentation and Separation of Trajectories

**Fig. 6:**
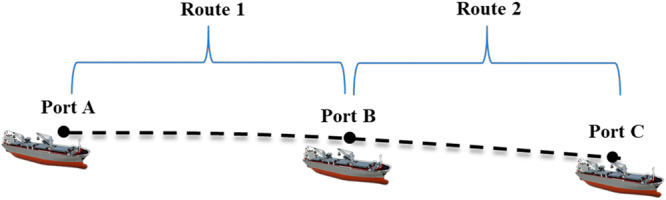
Vessel Trajectories Segmentation.

This step is designed to dissect the complete trajectory processed in step 3 of each vessel into distinct routes, guided by the identified sequence of events. For instance, if a vessel travels from Port A to Port B and then to Port C, its journey is segmented into two routes: Route 1 (Port A to Port B) and Route 2 (Port B to Port C), as illustrated in Fig. 6. This segmentation process categorizes ship trajectories into specific groups based on the stop or move label generated in step 1. The output at this step is a dataset containing information about all vessels’ routes, including carbon emissions, geographical details of each trajectory point, and the starting and ending ports of each route, along with their corresponding states.

Step 5: Calculating Route and State-Level Carbon Emissions

In scenarios where ports serve as both the end point of one route and the start point of another—for instance, Port B in Fig. 6—the emissions attributed to Port B (i.e., a stopping segment) are evenly distributed between Port B in Route 1 and Port B in Route 2. For each identified route, total carbon emissions are calculated by aggregating the emissions from all trajectory points within it, and then evenly allocated to the start and end states (i.e., ports). Subsequently, the comprehensive carbon emissions for each state are calculated by summing these emissions from all routes associated with that state. The ultimate output at this step is a dataset about route carbon emissions and state-level carbon emission.

**Fig. 7:**
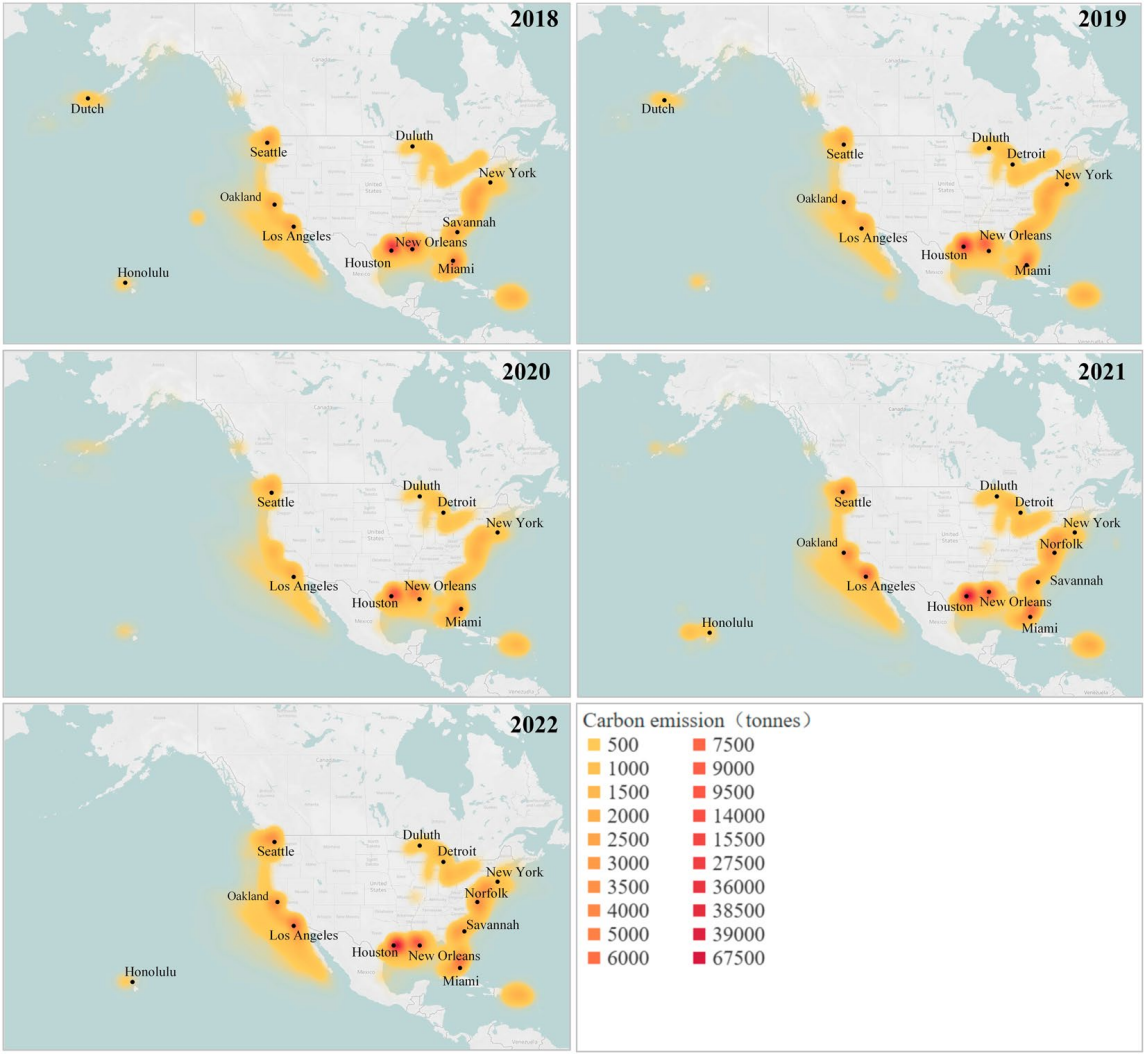
U.S. coastal carbon emissions from 2018 to 2022.

The heatmaps of carbon emissions are presented in Fig. 7, which were constructed using a uniform grid system with a spatial resolution of 1 km × 1 km. The figures illustrate that Houston, New Orleans, and Miami consistently exhibited high carbon emission levels. However, Dutch Harbor emerged as a carbon emission hot spot in 2018 and 2019, gradually diminishing from 2020 onward.

### Calculate the carbon transfer

**Fig. 8:**
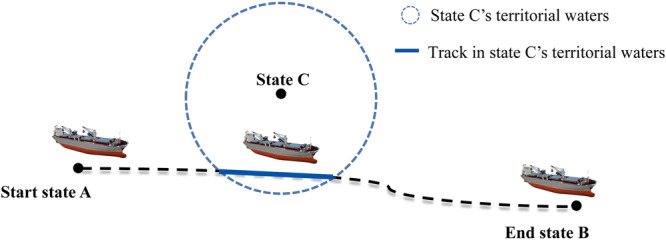
An example for carbon transfer description.

While prior research has delved into provincial-level carbon transfers^21–25^ and the spatio-temporal evolution of carbon transfers^26,27^, the exploration of maritime carbon transfer remains scarce. Our study presents a ground-breaking approach to defining and calculating carbon transfer within maritime activities. Consider the scenario illustrated in Fig. 8, where a vessel travels from a port in State A to a port in State B, passing through State C. In our methodology, the carbon emissions generated during the voyage are attributed to both the originating state (A) and the destination state (B). Furthermore, we introduce the concept that these emissions are also indirectly transferred to the intermediary state (C), through which the vessel transits. Consequently, emissions occurring within the boundaries of State C, depicted by the blue line in Fig. 8, are considered to be equitably transferred from States A and B. This approach enables a precise allocation and quantification of carbon transfers, facilitating a nuanced analysis of emission flows across different jurisdictions during maritime transport.

The algorithm for calculating carbon transfer adopts a holistic methodology that accounts for both vessel movements and stops. The process commences with MMSI data and geographical information. For each trajectory point labelled as “move,” the algorithm determines the corresponding state based on the point’s location.

As the ultimate output mentioned in the previous section, it comprises distinct ship routes, each linked to carbon transfer values for every visited state. This meticulous approach offers profound insights into carbon flow, providing a detailed understanding of emission distribution across different regions during maritime activities.

**Fig. 9:**
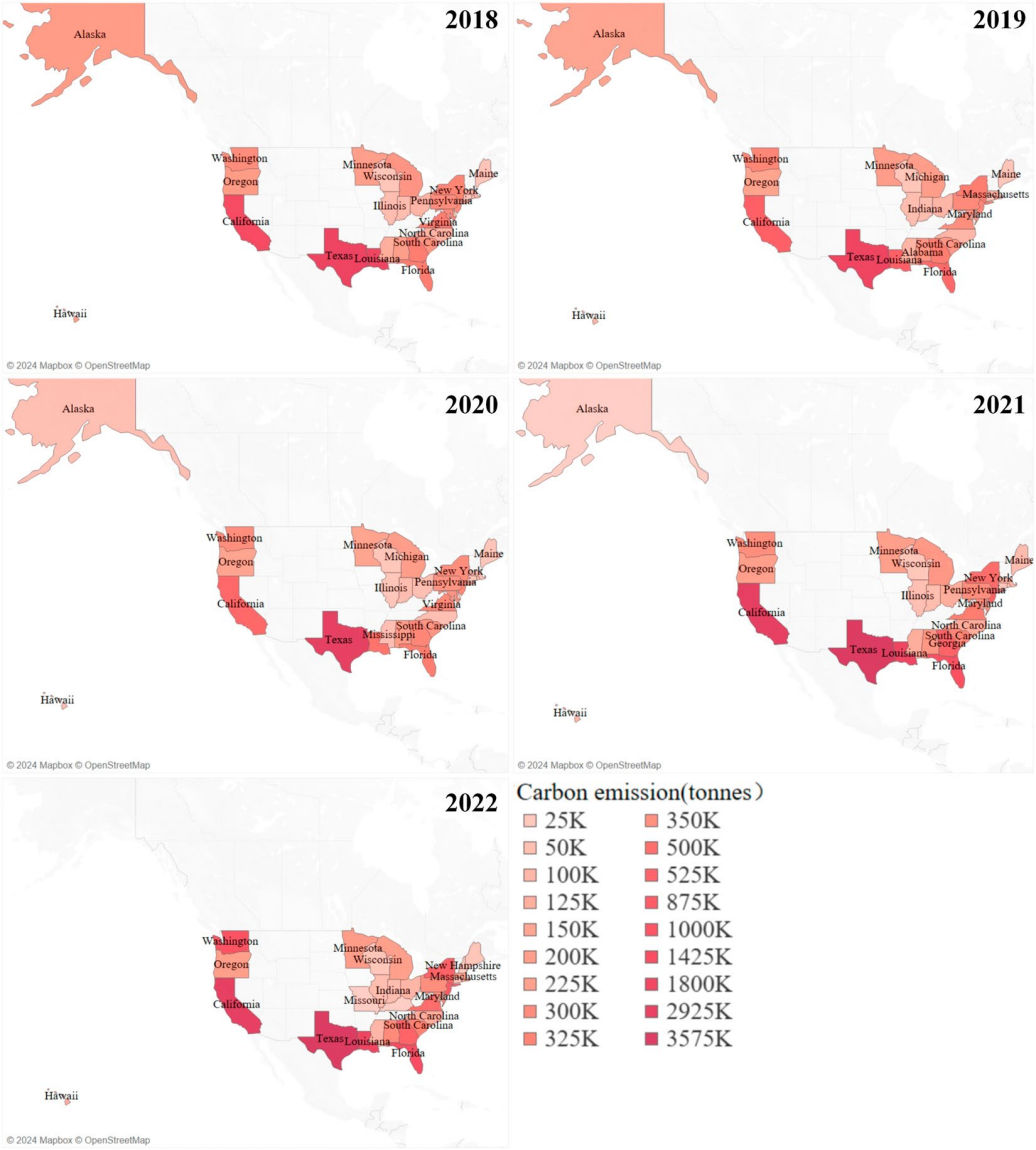
Carbon Emissions Generated by U.S. States from 2018 to 2022.

Figure 9 illustrates the aggregate carbon emissions produced by each U.S. state from 2018 to 2022. This total encompasses emissions originating within the state, those directed to other states, and emissions released into the high seas. Notably, Texas, California, and Washington consistently ranked as the top three carbon-emitting states.

**Fig. 10:**
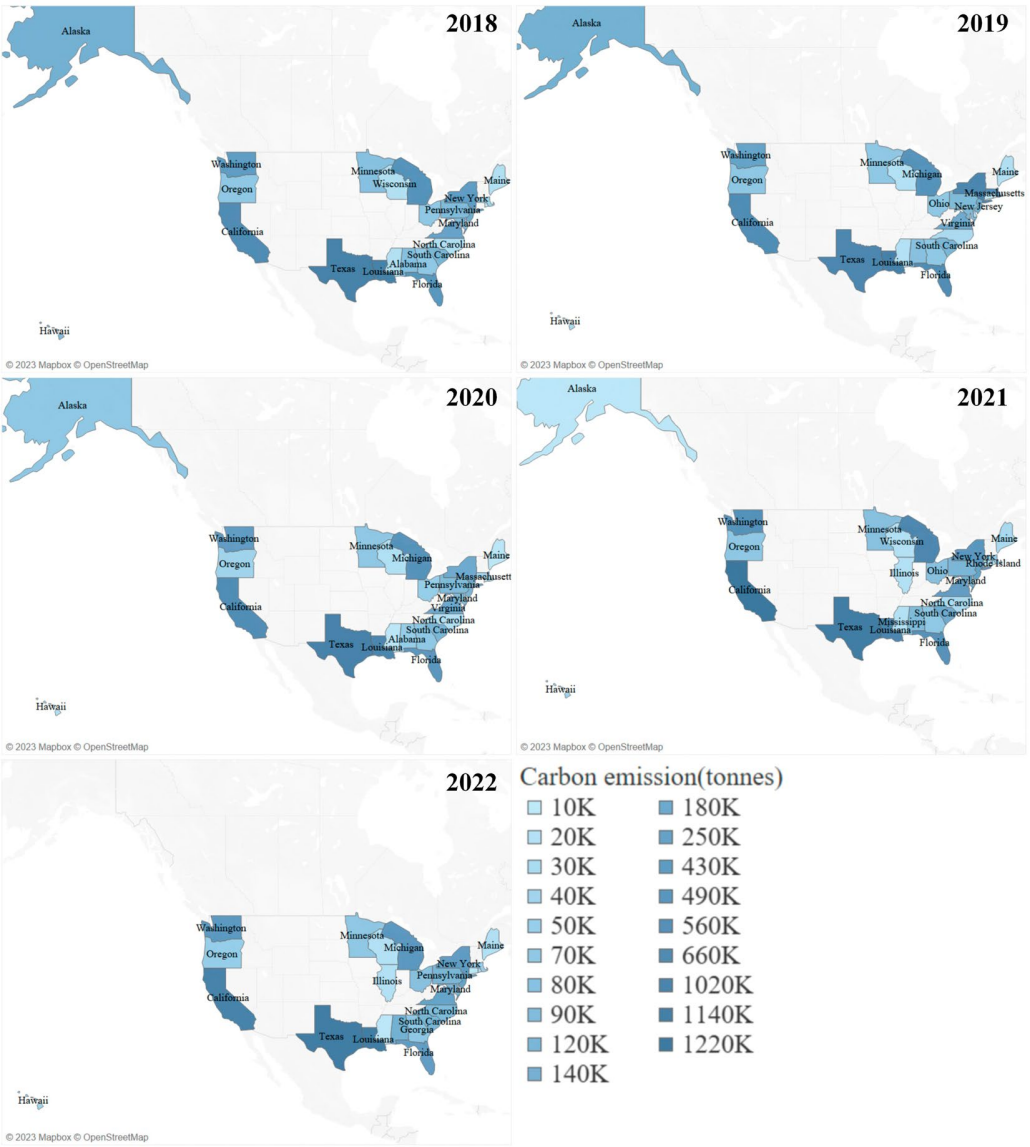
Carbon Emissions Received by U.S. States from 2018 to 2022.

In Fig. 10, we present the cumulative carbon emissions received by each U.S. state during the same period. This calculation incorporates emissions originating from the respective state and those received from other states. In addition to the top three states in carbon emission generation, Michigan and New York also registered substantial levels of carbon emissions received.

**Fig. 11:**
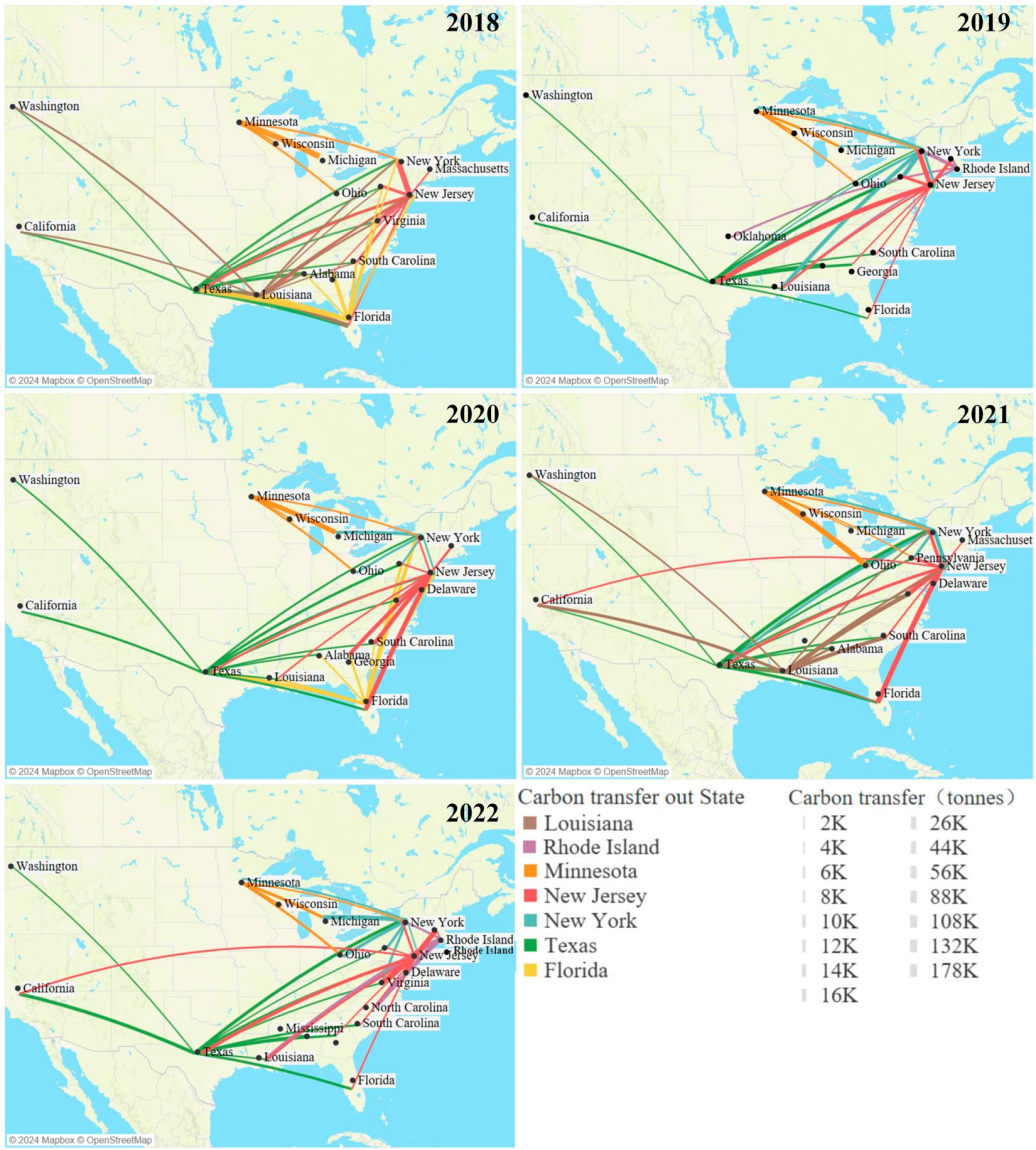
Carbon Transfer Paths for the Top 5 U.S. States with the Highest Transfer Volumes from 2018 to 2022.

Figure 11 illustrates the principal carbon transfer routes for the top 5 states with the highest volumes of carbon transfer. States manifest diverse carbon transfer patterns. Notably, emissions from Texas and New Jersey disperse across numerous destination states, whereas Michigan and New York predominantly channel carbon transfers to a limited set of states. Moreover, the primary carbon transfer routes changed over time. For instance, in 2018, the major routes included Louisiana to New York, Louisiana to California, Texas to Washington, and Texas to South Carolina. However, significant changes occurred, and by a later period, the major routes shifted, such as Minnesota to Pennsylvania, Minnesota to Ohio, Texas to New York, and New York to South Carolina.

### Calculate the overall carbon compensation

**Fig. 12:**
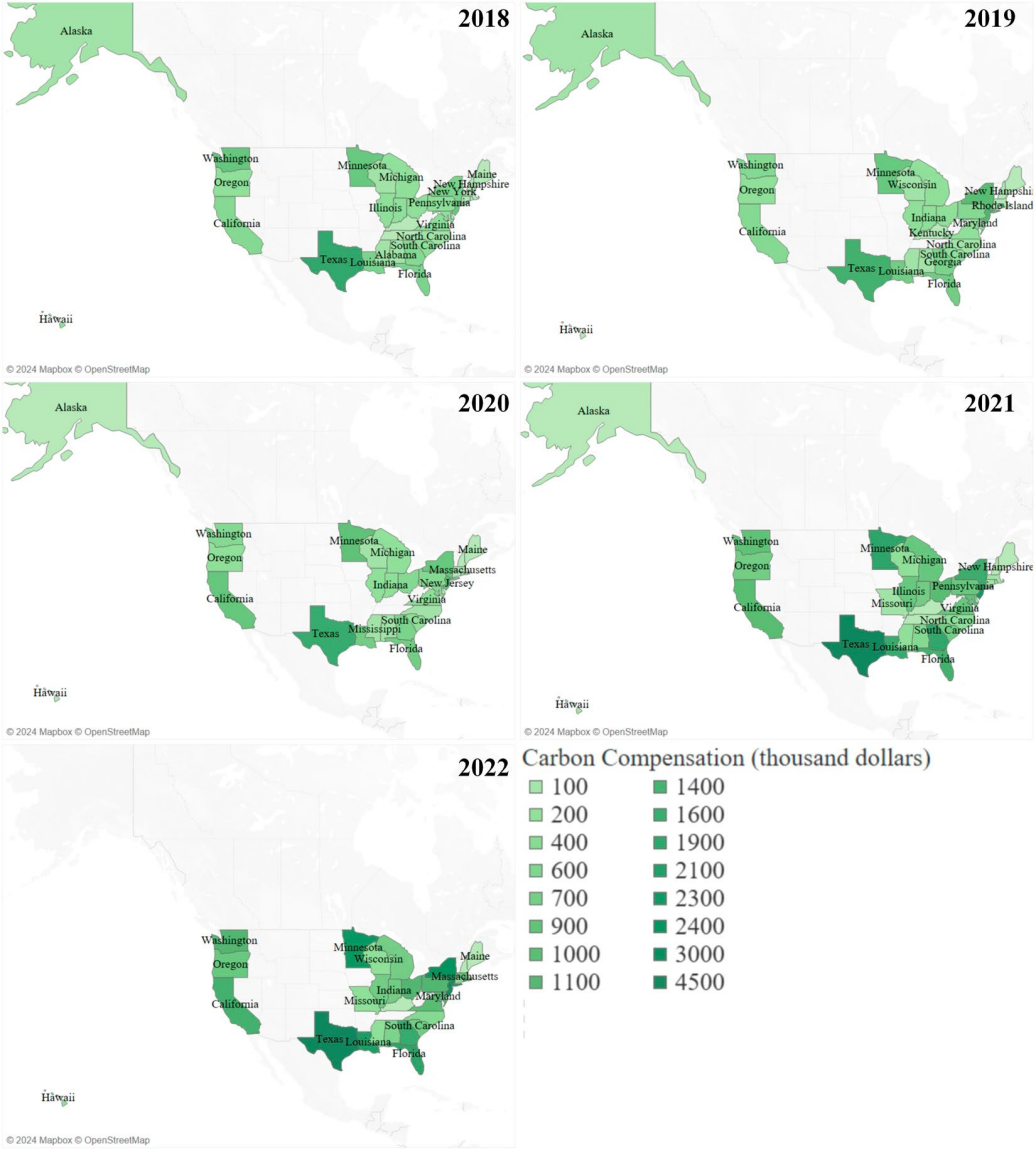
Carbon compensation in different states from 2018 to 2022.

Carbon prices fluctuated over the years 2018 to 2022, standing at $4.52/ton, $5.73/ton, $6.83/ton, $11.13/ton, and $14.44/ton^41^, respectively. Consequently, the yearly carbon compensation for each state is determined by multiplying the carbon price with the corresponding carbon transfer amount. Figure 12 visually represents the variation in carbon compensation across different states during the period from 2018 to 2022. Notably, Texas consistently emerged as the state receiving the highest carbon compensation.

## Data Records

Our calculation results are recorded in two files. “The Emissions of U.S. Freight Shipping Routes from 2018 to 2022.xlsx^42^” records the emissions of U.S. freight shipping routes from 2018 to 2022. The first and second columns of the file represent the year and MMSI numbers, respectively. The third, fourth, fifth and sixth columns indicate the port and state where a route started and arrived. The seventh column indicates the total emissions generated by each route, and the eighth and all subsequent columns indicate the emissions transferred to each state (including move stage emissions and stay stage emissions). “The Overall Carbon Transfer of U.S. Freight Ships from 2018 to 2022.xlsx^43^” illustrates the carbon transfer of U.S. freight ships from 2018 to 2022. The first column of the file represents the year. The second column of this file contains the states with carbon transfer out, and the first row lists the states with carbon transfer in.

## Technical Validation

### Accuracy analysis

In this section, we discuss the accuracy of the results. We have not found direct data on maritime freight transportation in the U.S., and we can only deduce it through indirect data. In our approach, we initially extract information about various types of freight vessels (i.e., general cargo ships and tankers) from AIS data within U.S. territorial waters to enhance reliability. Subsequently, a median filter was utilized to clean the data. Next, we developed carbon emission models for stop points and move points, which are calculated based on multiple factors including timestamp, location, speed, average power for both main and auxiliary engine, and other parameters. To enhance the precision of our carbon emission calculation, we transitioned from using Euclidean distance to the Haversine formula. Our results reveal that in 2018, total carbon emissions from U.S. freight vessels amounted to 14.41 million tons. According to the Inventory of U.S. Greenhouse Gas Emissions and Sinks 1990–2018, the national inventory that the U.S. prepares annually under the United Nations Framework Convention on Climate Change (UNFCCC), the carbon emission generated by freight ships was 13.9 million tons^44^. The error is 3.67%, which may be attributed to the utilization of average engine power. Considering the errors in the statistical process, the accuracy of the calculation method in this paper is relatively high. Furthermore, since our data is accurate for each vessel, using our method to calculate carbon emission is meaningful.

### Comparisons with existing emission databases

Since there are few databases on carbon emission from the U.S. maritime freight transport industry, the data in this article supplement existing data. Furthermore, the data in this article is accurate for the carbon emission of each vessel, and the data scale is more accurate.

### Supplementary information

Table S1

Table S2

supplementary information

## Data Availability

The data calculation was primarily performed by PyCharm Community Edition 2022.1.3, with Python 3.11 runtime environment (hosted on an Intel i5-1135G7 CPU @ 2.4 GHz, 16 GB RAM, and Windows 11 operating system). The custom code includes parameters that are integral to the dataset generation process. Specifically, the minimum stay time (“thre_sail”) is set to 1 hour, the minimum sailing time (“thre_stop”) is set to 1 hour, and the distance to the nearest port(“thre_shold”) is set to 0.2°. Researchers and interested parties can access the code through the provided, subject to the authors’ permission. The detailed codes can be found in Supplementary information.
