# Supplementary material for: Maritime Freight Carbon Emission in the U.S. using AIS data from 2018 to 2022
Source: Sci Data. 2024 May 25;11:542. doi: 10.1038/s41597-024-03391-0 (PMC11127944; doi:10.1038/s41597-024-03391-0)
Supplement: Supplementary file 3 — supplementary information [file 41597_2024_3391_MOESM3_ESM.pdf]

Content

Detailed codes 1

Detailed codes

```
import glob
import geopandas as gpd
from shapely.geometry import Point
import numpy as np
import pandas as pd


# -------------------------------------carbon emmsion-------------------------------------------#
def calculate_distance_haversine(lat1, lon1, lat2, lon2):
    # Convert latitude and longitude from degrees to radians
    lat1, lon1, lat2, lon2 = np.radians([lat1, lon1, lat2, lon2])

    # Haversine
    dlon = lon2 - lon1
    dlat = lat2 - lat1
    a = np.sin(dlat / 2) ** 2 + np.cos(lat1) * np.cos(lat2) * np.sin(dlon / 2) ** 2
    c = 2 * np.arcsin(np.sqrt(a))

    # The average radius of the earth; in nautical miles
    r = 3440
    distance = c * r
    return distance


# Calculate CO2
def calculate_co2(df, ship_type):
    co2_values = np.zeros(len(df))

    # Calculate the time difference between neighbouring rows (in h)
    df['TimeDiff'] = df['BaseDateTime'].diff().dt.total_seconds() / 3600

    # Calculate CO2 from the stay stage
    consecutive_stops = (df['Label'] == '1') & (df['Label'].shift() == '1')
    consecutive_stops_shifted_down = consecutive_stops.shift(-1, fill_value=False)
    # cargo_ship    if ship_type == 0 else tanker
    # emission coefficient I ==3.114,    the specific fuel consumption    SFC=213.1 g/kWh
    # pa = 1776kw for general cargo ships and pa = 1985kw    for oil tankers
    emission_rate_stop = 0.378 if ship_type == 0 else 0.423    # emission_rate_stop = pa * SFC *10** （-6）,
    co2_values[consecutive_stops] = df['TimeDiff'][consecutive_stops] * emission_rate_stop * 3.114    # in tonnes
    co2_values[consecutive_stops_shifted_down] = co2_values[consecutive_stops]/2
```

```
    # Calculate CO2 from the move stage
    moving_segments = df['Label'] == '0'
    speed_mean = (df['SOG'] + df['SOG'].shift(-1)) / 2
    distance = calculate_distance_haversine(df['LAT'], df['LON'], df['LAT'].shift(-1), df['LON'].shift(-1))
    mask = moving_segments & moving_segments.shift(-1)
    # ps = 9300kw for general cargo ships and ps = 9400kw  for oil tankers
    emission_rate_move = 1.982 if ship_type == 0 else 2.003  # # emission_rate_move = ps * SFC *10** （-6）
    co2_values[:-1][mask[:-1]] = emission_rate_move * distance[mask] / speed_mean[mask] * 3.114

    # Update CO2 column
    co2_values[(df['Label'] == '0') & (df['TimeDiff'].shift(-1) > 24)] = 0
    df['CO2'] = co2_values
    df.drop(columns=['TimeDiff'], inplace=True)
    df['CO2'] = df['CO2'].replace(np.inf, 0)  # Replacing infinity values with 0


# find nearby ports for stay
def find_nearby_ports(row, port_data, threshold):
    lat = row['LAT']
    lon = row['LON']
    nearby_ports = port_data[
        (abs(lat - port_data['Latitude']) <= threshold) &
        (abs(lon - port_data['Longitude']) <= threshold)
        ]
    port_name = ''  # Initialize port_name
    state = ''  # Initialize state
    # find nearest port
    if not nearby_ports.empty:
        if len(nearby_ports) == 1:
            port_name = nearby_ports['Main Port Name'].iloc[0]
            state = nearby_ports['state'].iloc[0]
        else:
            distances = nearby_ports.apply(lambda row: calculate_distance_haversine(lon, lat, row['Longitude'], row['Latitude']), axis=1)
            min_index = distances.idxmin()
            port_name = nearby_ports.loc[min_index, 'Main Port Name']
            state = nearby_ports.loc[min_index, 'state']
    return port_name, state
```

```python
# Data calculation for each mmsi vessel
def emmsion_process_csv(file_path, output_folder, port_data, threshold, usa_states, thre_sail, thre_stop, ship_type):
    df = pd.read_csv(file_path)
    df = df.sort_values('BaseDateTime')
    lat = df['LAT'].values
    lon = df['LON'].values
    # Time format conversion
    df['BaseDateTime'] = pd.to_datetime(df['BaseDateTime'])
    dat = (df['BaseDateTime'].values.astype(np.int64) // 10 ** 9).astype(int)

    # Initializes the state of each ship with 0 for motion and 1 for stay
    df['Label'] = '0'
    # ship state processing
    state = df['SOG'].isin([i / 10 for i in range(3)])

    # Initialize the list of stored stays
    stay_location = []    # stay location
    stay_start_ind = []    # stay start index
    stay_end_ind = []    # Stay end index
    stay_duration_hr = []    # Length of stay in hours

    # find the start index and end index of the stay in the continuous segment of the ship "state=true"
    start_ind = []
    end_ind = []
    start_ind = np.where(state & ~np.roll(state, 1))[0]
    end_ind = np.where(state & ~np.roll(state, -1))[0]

    # Calculate the stay time for each stay section and check that the minimum stay time requirements are met.
    for i in range(len(start_ind)):
        time_diff_hr = (dat[end_ind[i]] - dat[start_ind[i]]) / 3600
        if time_diff_hr >= thre_stop:
            stay_start_ind.append(start_ind[i])
            stay_end_ind.append(end_ind[i])
            # stay_duration_hr.append(time_diff_hr)
            # If so, the stop point:Label=1
            df.iloc[start_ind[i]:end_ind[i] + 1, df.columns.get_loc('Label')] = '1'

    # handling consecutive stops
    i = 0
    while i < len(stay_start_ind) - 1:
        current_start_ind = stay_start_ind[i + 1]
```

```
            prev_end_ind = stay_end_ind[i]
            time_diff_between_stays = (dat[current_start_ind] - dat[prev_end_ind]) / 3600

            # If the move time between two saty section is <thre_sail, then
            #        merge the two stays, the trajectory point between them label=1
            if time_diff_between_stays <= thre_sail:
                stay_end_ind[i] = stay_end_ind[i + 1]
                # stay_duration_hr[i] += stay_duration_hr[i+1] + time_diff_between_stays
                df.iloc[stay_start_ind[i]:stay_end_ind[i] + 1, df.columns.get_loc('Label')] = '1'
                stay_start_ind.pop(i + 1)
                stay_end_ind.pop(i + 1)
                # stay_duration_hr.pop(i+1)
            else:
                i += 1

    # Preserve the first and last rows of a stay
    drop_indices = [range(stay_start_ind[i] + 1, stay_end_ind[i]) for i in range(len(stay_start_ind))]
    drop_indices = [idx for sublist in drop_indices for idx in sublist]
    df.drop(drop_indices, inplace=True)

    # Save valid ship data
    if all(df['Label'] == '0') or len(df[df['Label'] == '1']) == 2:
        return

    df = df.reset_index(drop=True)

    # Calculate carbon emission
    calculate_co2(df, ship_type)

    # Find the state where the track point is located
    # Create point coordinates
    points = [Point(lon, lat) for lon, lat in zip(df['LON'], df['LAT'])]
    # Create a buffer of points
    buffered_points = [point.buffer(0.4) for point in points]
    # Create the GeoDataFrame
    gdf = gpd.GeoDataFrame(df, geometry=buffered_points, crs='EPSG:4326')
    gdf['State'] = ''
    # Determine the state # Update the state info columns
    points_in_states = gpd.sjoin(gdf, usa_states, how='left', predicate='intersects')
    gdf.loc[points_in_states.index, 'State'] = points_in_states['name']

    # Get the index of all rows where Label is 1
    label_1_indices = gdf.index[gdf['Label'] == '1']
```

```
        # update PortName
        gdf.loc[label_1_indices, 'PortName'] = gdf.loc[label_1_indices].apply(
            lambda row: find_nearby_ports(row, port_data, threshold)[0], axis=1)
        # update State
        gdf.loc[label_1_indices, 'State'] = gdf.loc[label_1_indices].apply(
            lambda row: find_nearby_ports(row, port_data, threshold)[1], axis=1)

        # Save the results to a CSV file
        output_filename = os.path.basename(file_path)
        output_file_path = os.path.join(output_folder, output_filename)
        gdf.drop('geometry', axis=1).to_csv(output_file_path, index=False)


def main_emmsion():
    # Processing ship data
    # Data processing, minimum stay time, minimum sailing time
    thre_sail = 1
    thre_stop = 1

    # Find the port and read the port file
    port_data = pd.read_csv('usa_port_data.csv')
    # Set the port proximity threshold
    threshold = 0.2

    # Read the Shapefile file that contains U.S. state borders
    usa_states = gpd.read_file('ne_110m_admin_1_states_provinces')
    # Set up a coordinate reference system for state boundary data
    usa_states = usa_states.to_crs('EPSG:4326')

    # Handling cargo ships
    for i in range(2018, 2023):
        folder_path = f'E:/{i}/cargo_mmsi'    # Data extracted and preprocessed according to
mmsi
        output_folder = f'E:/{i}/cargo_stop'
        os.makedirs(output_folder, exist_ok=True)
        file_list = glob.glob(os.path.join(folder_path, '*.csv'))
        ship_type = 0
        for file_path in tqdm(file_list, desc='Processing'):
            emmsion_process_csv(file_path, output_folder, port_data, threshold, usa_states,
thre_sail, thre_stop,
                                ship_type)
        print(f'{i}year cargo ship calculation completed')

    # Handling tanker
```

```
    for i in range(2018, 2023):
        folder_path = f'E:/{i}/tanker_mmsi'    # Data extracted and preprocessed according to mmsi
        output_folder = f'E:/{i}/tanker_stop'
        os.makedirs(output_folder, exist_ok=True)
        file_list = glob.glob(os.path.join(folder_path, '*.csv'))
        ship_type = 1
        for file_path in tqdm(file_list, desc='Processing'):
            emmsion_process_csv(file_path, output_folder, port_data, threshold, usa_states, thre_sail, thre_stop,
                                ship_type)
        print(f'{i}year tanker calculation completed')


if __name__ == "__main__":
    main_emmsion()

# -------------------------------------------Carbon transfer-----------------------------------------#
import pandas as pd
import os
import numpy as np
from tqdm import tqdm


def transfer_process_csv(file_path):
    df = pd.read_csv(file_path)
    # Find the starting index of the stay
    state = df['PortName'].notnull()
    # print("State array:", state)
    start_ind = [i for i in range(1, len(state)) if state[i] and not state[i - 1]]
    end_ind = [i for i in range(len(state) - 1) if state[i] and not state[i + 1]]
    # print("Start indices:", start_ind)
    # print("Start indices:", end_ind)

    # Lists to store stay information
    mmsi_list = []
    start_port_list = []    # Departure port
    start_state_list = []    # State of departure port
    end_port_list = []    # Arrival port
    end_state_list = []    # State of destination port
    generate_co2_list = []    # Total CO2 produced by the trip

    for i in range(len(start_ind) - 1):
        mmsi = df['MMSI'].iloc[end_ind[i]]
```

```
        start_port = df['PortName'].iloc[end_ind[i]]
        start_state = df['State'].iloc[end_ind[i]]
        end_port = df['PortName'].iloc[start_ind[i + 1]]
        end_state = df['State'].iloc[start_ind[i + 1]]

        # Calculate the total emissions per flight
        co2_sum = df['CO2'].iloc[end_ind[i]:start_ind[i + 1] + 1].sum()

        mmsi_list.append(mmsi)
        start_port_list.append(start_port)
        start_state_list.append(start_state)
        end_port_list.append(end_port)
        end_state_list.append(end_state)
        generate_co2_list.append(co2_sum)

    # Create a DataFrame to store the result
    result_df = pd.DataFrame({'mmsi': mmsi_list,
                              'start_port': start_port_list,
                              'start_state': start_state_list,
                              'end_port': end_port_list,
                              'end_state': end_state_list,
                              'generate_co2': generate_co2_list})
    # Calculate the CO2 transfer between states in a year
    for i in range(len(start_ind) - 1):
        states_visited = df.loc[end_ind[i]:start_ind[i + 1] + 1, 'State'].unique()
        for state in states_visited:
            co2_transfers = df.loc[(df['State'] == state) &
                                   (end_ind[i] <= df.index) & (df.index <= start_ind[i + 1]),
'CO2'].sum()
            result_df.loc[i, f'to_{state}'] = co2_transfers

    return result_df


def main_transfer():
    for i in range(2018, 2023):
        # Create an empty DataFrame to store all processed data
        all_data = pd.DataFrame()

        folder_path1 = f'E:/{i}/cargo_stop'
        folder_path2 = f'E:/{i}/tanker_stop'
        # Get all files from both folders
        files_in_folder1 = [os.path.join(folder_path1, file) for file in os.listdir(folder_path1) if
                            file.endswith('.csv')]
```

```
        files_in_folder2 = [os.path.join(folder_path2, file) for file in os.listdir(folder_path2) if file.endswith('.csv')]
        # Combine the files from both folders
        all_files = files_in_folder1 + files_in_folder2

        # Iterate over each file
        for file_path in tqdm(all_files, desc=f'Processing {i}'):
            # carbon transfer
            result_df = transfer_process_csv(file_path)
            all_data = pd.concat([all_data, result_df])

        # Reset index and save to CSV
        all_data.reset_index(drop=True, inplace=True)
        all_data.to_csv(f'E:/{i}/ship_routes.csv', index=False)

        # Initializes the state and co2 lists
        states = []
        co2_values = []

        # Iterate over each trace in all_data
        for _, row in all_data.iterrows():
            start_state = row['start_state']
            end_state = row['end_state']
            # Calculate carbon emissions for this trajectory (average carbon emissions = generate_co2/2)
            average_co2 = row['generate_co2'] / 2

            # Add start_state to the list If it is not in the states list
            if start_state not in states:
                states.append(start_state)
                co2_values.append(average_co2)
            else:
                # The carbon emission of the trajectory is added to the corresponding position
                index = states.index(start_state)
                co2_values[index] += average_co2

            # Do the same for end_state
            if end_state not in states:
                states.append(end_state)
                co2_values.append(average_co2)
            else:
                index = states.index(end_state)
                co2_values[index] += average_co2
```

```python

        # Merge the state and co2 lists into a single DataFrame
        state_co2_df = pd.DataFrame({'State': states, 'co2': co2_values})

        # Extract start_state, generate_co2, and subsequent columns
        start_cols = [col for col in all_data.columns if col.startswith('generate_co2') or col.startswith('to_')]
        df_start = all_data[['start_state'] + start_cols].rename(columns={'start_state': 'state'})

        # Extract end_state, generate_co2, and subsequent columns
        end_cols = [col for col in all_data.columns if col.startswith('generate_co2') or col.startswith('to_')]
        df_end = all_data[['end_state'] + end_cols].rename(columns={'end_state': 'state'})

        # Merge the above DataFrames
        df_combined = pd.concat([df_start, df_end], axis=0)
        grouped_data = df_combined.groupby('state')

        # Create an empty DataFrame to store the result
        aggregated_data = pd.DataFrame()

        for _, group in grouped_data:
            state = group['state'].iloc[0]
            emmsion_co2 = round(group['generate_co2'].sum() / 4, 2)
            result_dict = {'State': state, 'emmsion_co2': emmsion_co2}

            # Extract all transfer columns(to_state) after state and add their values to the result_dict
            transfer_cols = [col for col in group.columns if col.startswith('to_')]
            for col in transfer_cols:
                transfer_sum = round(group[col].sum() / 4, 2)
                result_dict[col] = np.nan if transfer_sum == 0 else transfer_sum

            aggregated_data = pd.concat([aggregated_data, pd.DataFrame([result_dict])])

        aggregated_data.reset_index(drop=True, inplace=True)
        # store results
        aggregated_data.to_csv(f'E:/{i}/ship_transfer.csv', index=False)


if __name__ == "__main__":
    main_transfer()
```
